# Mesenchymal stem cells in cancer progression and anticancer therapeutic resistance

**DOI:** 10.1186/s12935-021-02300-4

**Published:** 2021-11-04

**Authors:** Xiuyun Xuan, Chunxia Tian, Mengjie Zhao, Yanhong Sun, Changzheng Huang

**Affiliations:** 1 Department of Dermatology, Union Hospital, Tongji Medical College, Huazhong University of Science and Technology, Wuhan, 430022 Hubei China; 2 Department of Cardiology, Hubei Provincial Hospital of TCM, Wuhan, 430022 Hubei China; 3 Department of Dermatology, Zhongnan Hospital, Wuhan University, Wuhan, 430022 Hubei China; 4 Department of Dermatology, Second Affiliated Hospital, Zhejiang University School of Medicine, Hangzhou, 310009 Zhejiang China

**Keywords:** Mesenchymal stem cells, Cancer progression, Therapeutic resistance, Tumor microenvironment, Cytokine

## Abstract

Increasing evidence indicates that the tumor microenvironment appears to play an increasingly important role in cancer progression and therapeutic resistance. Several types of cells within the tumor stroma had distinct impacts on cancer progression, either promoting or inhibiting cancer cell growth. Mesenchymal stem cells (MSCs) are a distinct type of cells that is linked to tumor development. MSCs are recognized for homing to tumor locations and promoting or inhibiting cancer cell proliferation, angiogenesis and metastasis. Moreover, emerging studies suggests that MSCs are also involved in therapeutic resistance. In this review, we analyzed the existing researches and elaborate on the functions of MSCs in cancer progression and anticancer therapeutic resistance, demonstrating that MSCs may be a viable cancer therapeutic target.

## Background

Mesenchymal stem cells (MSCs) are a kind of multipotent cells with a high differentiation potential and self-renewal ability, making them a promising regenerative medicine population [1]. MSCs are easy to acquire and may be separated from a wide range of tissues. The major source of MSCs is believed to be bone marrow (BM), although the content is minimal, accounting for just 0.001–0.01% of total nucleated cells [2]. In the BM, MSCs support haemopoiesis and regulate immune activity [3]. Moreover, MSCs may also be isolated from adipose tissue, which comes from people who have had liposuction. Adipose tissue-derived stem cells serve a crucial role in reconstructive or tissue engineering medicine [4]. In addition to the two sources of MSCs mentioned above, MSCs can be also effectively extracted from other tissues, such as umbilical cord [5], umbilical cord blood [6], amniotic membrane [7], placenta [8], peripheral blood [9], muscle [10] and lung [11]. MSCs have a variety of functions in these organs, including contributing to organ homeostasis and tissue-specific healing [12]. In addition, MSCs have three basic characterizations. To begin, morphological characterization of MSCs revealed that they are a heterogeneous cell population with numerous cell subsets that are spindle-form fibroblast-like, flattened, or spherical in shape [13–15]. Second, the functional and differentiation characteristics of MSCs indicated that, when subjected to the appropriate stimuli, they may differentiate into a number of cell types (such as fibroblasts, adipocytes, chondrocytes and osteoblasts) and perform a variety of roles [16]. Third, immunophenotypic characterization of MSCs exhibited that they express the cell surface markers CD105, CD90 and CD73 but lack CD45, CD34, CD14, CD11b, CD79a or CD19, as well as HLA-DR [17]. Moreover, MSCs are also said to have immune-suppressive properties [1]. MSCs modulate immunity by producing cytokines and regulating several immune cells’ function [18]. For example, MSCs have been shown to be effective in treating graft disease versus host disease [19] and some autoimmune diseases [20]. In addition, MSCs also have the ability to migrate to tumor and inflammatory areas. Several chemokines and related receptors may be involved in the process of MSC migration, including growth factors (i.e., EGF, SCF, PDGF, HGF and IGF-1 [21, 22]), angiogenic factors (i.e., VEGF, HIF1α and βFGF [23, 24]), chemokines (i.e., CCL2, CCL5, CCL22 and CXCL12 [22, 25]), inflammatory factors and other cytokines (i.e., TNFα, TGFβ, IL-1β, IL-8 [26–29]). Recently, it is reported that cancer cell-derived exosomes (Exo) have been shown to regulate the migration and homing of MSCs by inducing the expression of circular RNAs. In gastric cancer, for example, after MSCs were treated with adenocarcinoma gastric cells cell-derived Exo, elevated hsa_circ_0004303 in MSCs promoted the biological activities of MSCs via the miR-148a-3P/ALCAM axis [30]. These findings showed that a variety of mediators may aid MSC migration to various types of tumor locations, therefore promoting or inhibiting tumor growth. Because of their homing ability, MSCs have been successfully utilized to treat spinal cord injury, damaged kidneys, diabetes, myocardial infarction, and bone injury [17]. Furthermore, MSCs have been shown to have the capacity to stimulate angiogenesis during the process of ischemia [31] and wound healing [32]. In summary, MSCs perform a variety of vital functions under physiological and pathological circumstances, and play crucial roles in the treatment of several diseases. In addition, MSCs have been indicated to also have a positive or negative effect on cancer pathogenesis.

In this review, we gathered related researches and discussed the functions of MSCs in cancer progression and anticancer therapeutic resistance, which may provide potent targets for cancer and find new improvements for therapeutic resistance.

## MSCs in cancer progression

Tumor microenvironment (TME) heterogeneity is a significant component that impacts tumorigenesis. TME is a complex ecosystem that comprises stromal cells, extracellular matrix components, and secreted factors [33]. Among of them, stromal cells in TME incorporate that adipocytes, endothelial cells, cancer-associated fibroblasts, immune cells and MSCs [34]. MSCs, in particular, have a strong tropism to tumor locations, accelerating or slowing cancer growth. However, the exact mechanism is not clear. Then we will discuss the role of MSCs in cancer pathogenesis.

### MSCs promote cancer progression

TLRs (Toll-like Receptors) are found on a variety of cell types, including MSCs. TLRs have the capacity to detect ‘‘danger” signals, and their activation attracts a variety of cells to the damaged region, including immune cells and MSCs. Interestingly, TLR3 activation resulted in MSCs secreting some factors with mostly tumor supportive immunosuppressive effect (such as IL1RA and IL10), while TLR4 stimulation leaded to MSCs producing inflammatory and proapoptotic factors (such as IL17, GM-CSF, and TRAIL). TLR4-primed MSCs are called MSC1 that exhibited an antitumorigenic impact, while TLR3-primed MSCs are called MSC2 that had a tumor-supportive function [35]. In addition, according to Ruth and colleagues, MSC1 inhibits tumor growth while MSC2 increases tumor growth and metastasis in vivo and in vitro [36]. Interestingly, the type of TLR agonist exposure to MSCs help the switch between MSC1 and MSC2. In other words, TLR4 agonists polarize MSCs towards pro-inflammatory MSC1 which is crucial for early injury responses, whereas TLR3 agonist exposure will polarize MSCs towards immunosuppressive MSC2 which is necessary for helping to heal tissue injury. Maybe it can help explain why MSCs have diverse roles in various cancer types.

Notably, MSCs have been shown to inhibit the anti-tumor immune response, including innate and adaptive immune responses, by secreting a variety of soluble factors and mediators (such as prostaglandin E2 (PGE2), IFNγ, IL-4, indoleamine 2,3-dioxygenase (IDO), TGF-β1, IL-6) and interacting with diverse immune cell types (such as T cell, B cells, macrophages, dendritic cells, NK cells and neutrophils) [37]. In adaptive immune responses, MSCs reduced T cell activation and proliferation. MSCs secreted PGE2, then bind to prostaglandin EP2 and EP4 receptors to reprogram macrophages by producing anti-inflammatory factor IL-10, which thus inhibit T cells [38]. Moreover, MSCs elicited Th2-polarized immune response. In other words, inflammatory T cells and associated cytokines (Th1 cells-IFNγ) were reduced, whereas anti-inflammatory T cells and related cytokines (Th2 cells-IL4) were elevated [39]. In addition, MSCs have been shown to block T cell activation by releasing immunosuppressive TGF-β1, which bind to glycoprotein a repetitions predominant (GARP) expressed on MSCs [40]. Furthermore, through degrading tryptophan, IDO produced by MSCs was able to suppress allogeneic T-cell responses [41]. Notably, in naive CD4^+^ T cells, tryptophan catabolism triggered the development of FOXP3-positive regulatory T cells (Treg) [42]. These cells reduced anti-tumor immunity by suppressing effector T cell responses. Recently, a novel mechanism by which MSCs regulate the immune system was discovered. It is that MSCs recruited myeloid-derived suppressor cells (inhibitory immune cells) in a CCL2-dependent way, reducing anti-cancer T cell activity even more [43]. In addition to T cells in adaptive immune response, MSCs can also suppress B cell functions. MSCs produced humoral substances that inhibited B cell activity by suppressing B cell terminal differentiation [44]. IFNγ-activated MSCs increased the expression of galectin-9, which inhibited antigen triggered immunoglobulin release and slowed B cell proliferation [45]. Taken together, MSCs have strong inhibitory effects on adaptive immune response, which is extensively exploited by cancer cells within TME. In addition to suppression of adaptive immune response, MSCs also inhibited innate immune cells to weaken primary anti-cancer immune responses. PGE2 and IL-6 produced by MSCs suppressed NK cell functions. And, MSCs primarily inhibited IFN-γ production in NK cells, thus weakening their anti-cancer activity [46]. Moreover, anti-cancer activities are inextricably linked with dendritic cells (DCs), which act to present antigens. It has been shown that DCs maturation and function were inhibited in the presence of PGE2 generated by MSCs [47]. And, MSCs suppressed development and function of monocyte-derived DCs with costimulatory molecules CD80/CD86 expression reduced, limiting allogeneic T cell allostimulatory capacity [48]. In addition, within the TME, macrophage activity was reduced directly by MSCs. MSC-derived conditioned medium (CM) has been shown to impair macrophage phagocytic activity, further decreasing anti-cancer immunity [49]. And, MSC-derived PGE2 induced a transition from inflammatory M1 macrophages to a pro-tumorigenic alternatively activated M2 state, which was accompanied by increased levels of immune-inhibitory IL-10 [50]. Furthermore, neutrophils activity was also influenced by MSCs. In breast tumor model, CD11b^+^Ly6G^+^ neutrophils were trained to acquire immunosuppressive activity following coculture with MSCs, suppressing T cell proliferation in vitro, and enhancing tumor progression in vivo [51]. Similarly, in gastric cancer, the chemotaxis, survival and activation of neutrophils were regulated via IL6-STAT3-ERK1/2 signaling, thus supporting tumor progression [52]. Taken together, these data above indicated that MSCs were able to suppress the anti-tumor immune response, therefore prompting tumor growth.

Moreover, MSCs were able to stimulate cancer cell growth and angiogenesis. In breast and prostate tumors, for example, MSCs raised the amounts of pro-angiogenic factors, such as MIP-2, VEGF, TGF-β and IL-6. These factors induced tumor cells proliferation and angiogenesis, thereby increasing the pace of solid tumor development in vitro and in vivo [53]. Similarly, in hepatocellular carcinoma, Li et al. discovered that the level of microvessel density and TGFβ1 mRNA were considerably enhanced, but Smad7 mRNA expression was inhibited in the MSC treated group. Their study indicated that MSCs may stimulate angiogenesis via the TGFβ1/Smad pathway [54]. Recently, Yuan et al. discovered that LncRNA H19 is implicated in MSC-mediated angiogenesis [55]. They found that LncRNA H19 knockdown in MSCs suppressed angiogenesis by associating with histone methyltransferase EZH2 and activating the angiogenesis inhibitor gene VASH1, reducing angiogenesis factors secretion and increasing angiogenesis inhibitors production.

In addition, MSCs promoted cancer cells metastasis, thus hastening tumor development. When breast cancer cells were directly co-cultured with MSCs, they demonstrated substantial overexpression of oncogenes (NCOA4, FOS), proto-oncogenes (FYN, JUN), and EMT specific markers, as well as shape and growth pattern changes, resulting in breast cancer metastasis [56]. Notably, cancer stem cells (CSCs) are particularly important in tumor metastasis. MSCs were indicated to prompt CSCs proliferation by producing several tumor-supportive mediators, thus making tumor spread and growth easier [57]. In addition, mesenchymal niche may be involved in cancer metastasis. Emerging evidences suggested that MSCs can move to tumor locations, including primary and pre-metastatic sites [58]. Tumor-secreted factors may go to neighboring tissues [59], where they attract MSCs to help build up the mesenchymal niche, which encourages cancer cell migration. In breast cancer, cancer cells induce production of CCL5 (also called RANTES) from MSCs via interacting with CCR5, increasing cancer cell motility, invasion, and metastasis in vitro and in vivo [60].

Furthermore, MSCs were also able to prevent tumor cells from undergoing apoptosis. As is all known, hypoxia, malnutrition, and inflammation are all recognized to have roles in tumor pathogenesis. MSCs sustain their self-survival in these settings via autophagy and the release of numerous pro-survival or anti-apoptotic factors, such as VEGF, bFGF, PDGF, TGF-β, SDF-1α, HGF, and Nitric oxide (NO) [61]. VEGF and bFGF, for example, can boost Bcl-2 expression [62, 63], while PDGF and TGF-β can boost VEGF and bFGF gene expression [64]. SDF-1α has been shown to protect leukemia cells against spontaneous apoptosis [65]. And, HGF improved the angiogenic and anti-apoptotic effects [66]. Moreover, NO was thought to be a dual-function apoptotic regulator. In short, at large doses, NO is proapoptotic, whereas at low doses, it is antiapoptotic [67].

Additionally, MSCs support tumor growth by changing their metabolic state. In lymphoblastic leukemia, MSCs-derived PGE2 activated cAMP-PKA signaling in tumor blasts and inhibited the tumor-suppressive function of wild type p53, thus promoting leukaemogenesis [68]. When exposed to oxidative stress in the TME, MSCs can produce lactate, and when cancer cells absorb lactate, their migration is enhanced by producing ATP [69]. Particularly, MSCs have been observed to differentiate into CAFs in vitro, which could drive tumor heterogeneity and play a crucial role in cancer progression and drug resistance [70]. In addition, increasing evidence has shown that noncoding RNAs participate in tumorigenesis and drug resistance [71–73]. A recent study showed that in gastric cancer, TGF-β1 secreted by MSCs activated the SMAD2/3 pathway and supported cancer progression through the lncRNA MACC1-AS1/miR-145-5p/fatty acid oxidation (FAO) axis in cancer cells [74]. Moreover, in triple-negative breast cancer, MSCs strongly induced the regulation of LINC01133 in adjacent tumor cells, which increases the propagation of CSC-like phenotypic characteristics and therefore strengthens cancer cell growth [75].

**Fig. 1:**
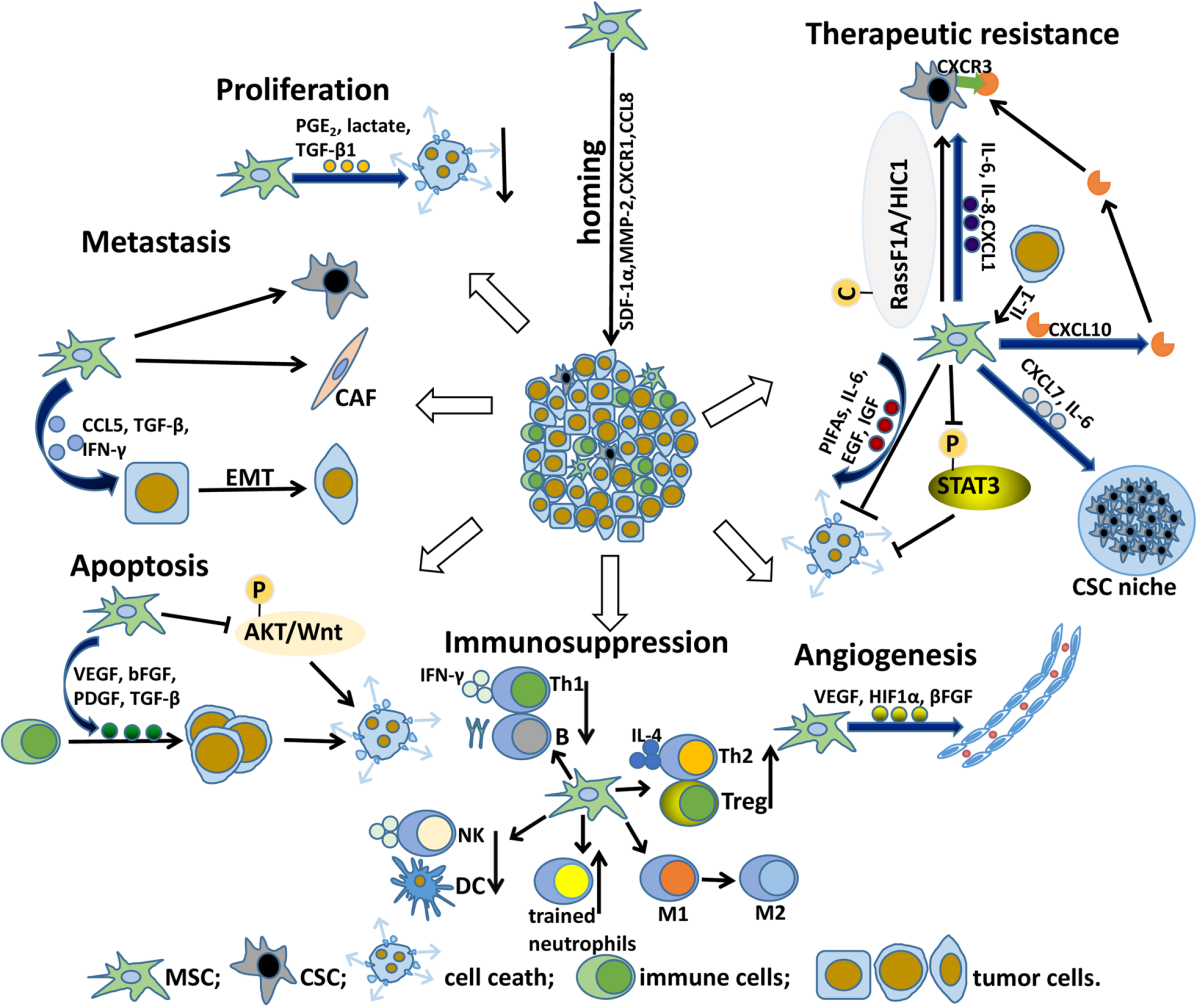
Roles of MSCs in cancer progression and therapeutic resistance. MSCs are well known for homing to tumor sites, where they play crucial roles in cancer parthenogenesis and therapeutic resistance. Several factors are involved in the process, such as SDF-1α, MMP-2, CXCR1 and CCL8. Proliferation: mediators (i.e., PGE_2_, lactate and TGF-β1) secreted by anti-inflammatory MSCs inhibited tumor cell death to further support tumor growth. Metastasis: MSCs are able to transform into CSCs or CAFs, which drives tumor heterogeneity. Moreover, several factors (i.e., CCL5, TGF-β and IFN-γ) produced by MSCs induced EMT, which contributed to metastasis. Apoptosis: several chemokines (i.e., VEGF, bFGF, PDGF and TGF-β) by MSCs recruited immune cells to tumor sites, thereby inducing anticancer immunity. In contrast, MSCs reduced cancer cell growth via paracrine inhibition of AKT and Wnt signaling pathways, which contribute to cancer cell proliferation. Immunosuppression: MSCs inhibited the anti-tumor immune response, including innate and adaptive immune responses. In adaptive immune responses, MSCs elicited Th2-polarized immune response, and indirectly triggered the development of Treg cells. Moreover, MSCs inhibited antigen triggered immunoglobulin release and slowed B cell proliferation. In innate immune responses, MSCs inhibited IFN-γ production in NK cells, and DCs maturation and function. Moreover, MSC induced a transition from inflammatory M1 macrophages to a pro-tumorigenic alternatively activated M2 state. In addition, trained neutrophils acquired immunosuppressive activity. Angiogenesis: MSCs secreted angiogenic factors (i.e., VEGF, HIF1α and βFGF) to promote angiogenesis, which contributes to cancer progression. Therapeutic resistance: paracrine or systematically secreting molecules (i.e., PIFAs, IL-6, EGF and IGF) by MSCs reduced tumor cell death, thereby inducing therapeutic resistance. In contrast, MSCs also suppress cancer pathogenesis and sensitize cancer cells to therapy by blocking the STAT3 pathway. MSCs support the CSC niche to further promote resistance to therapeutics, including CXCL7 and IL-6. In addition, several tumor suppressor genes in MSCs, such as RassF1A and HIC1, were methylated, thus indicating that MSCs transform into CSCs. Notably, tumor cells released IL-1, which induced MSCs to secrete several mediators (i.e., IL-6, IL-8 and CXCL1). These mediators together induce the formation of CSCs. Furthermore, MSCs significantly increased CXCL10 expression, which further promoted CSC proliferation when the CXCL10 receptor (CXCR3) was overexpressed on CSCs. As a result, they altogether increased tumor growth and enhanced therapeutic resistance

These findings revealed that MSCs support cancer progression via distinct mechanisms (Table 1; Fig. 1). Targeting MSCs represents a potential strategy for the treatment of cancer.

**Table 1:** Roles of MSCs in cancer progression

In vitro or in vivo	Source of MSCs	In vivo model	Cancer types	Functions	Mechanisms	References
In vitro and in vivo	Murine BM and human BM	Nude mice with 4T1 tumor and nude mice with DU145 tumor	Breast cancer; prostate cancer	Promoting tumor angiogenesis	Increasing the expression of markers associated with neovascularization (MIP-2, VEGF, TGF β and IL-6)	[53]
In vivo	Human BM	Nude mice with MHCC97-H tumor	HCC	Promoting tumor angiogenesis	Promoting tumor angiogenesis partly via the action of TGF β1	[54]
In vitro and in vivo	Lung cancer tissue and normal lung tissue	NOD-SCID-common-γ-KO (NSG) mice with lung tumors	Lung cancer	Promoting tumor metastasis	Inducing the expression of aggressive phenotype-associated genes (GREM1, LOXL2, ADAMTS12 and ITGA11)	[142]
In vitro	Human BM	–	Breast cancer	Promoting cancer cells metastasis	Upregulating of oncogenes, proto-oncogenes and EMT specific markers	[56]
In vitro and in vivo	Human BM	Athymic female nude mice and NOD/SCID mice with breast tumor	Breast cancer	Enhancing the motility, invasion and metastasis of cancer cells	Stimulating MSCs to secrete CCL5	[60]
In vitro and in vivo	Human BM	NOD/SCID mice with breast tumor	Breast cancer	Boost CSCs proliferation by producing IL-6 and CXCL7	Facilitating the tumor metastasis and growth	[57]
In vitro	Human BM	–	Acute lymphoblastic leukemia	Changing tumor cell metabolic state	Suppressing wild-type p53 via PGE2-cAMP-PKA signaling pathway	[68]
In vitro and in vivo	Human AT	BALB/C nude mice with PDAC	PDAC	promoting cancer progression	Differentiating into CAFs	[70]
In vitro and in vivo	Human BM	BALB/C nude mice with MKN45 tumor	Gastric cancer	Promoting cancer progression	TGF-β1 secreted by MSCs activated SMAD2/3 pathway and supported cancer progression through lncRNA MACC1-AS1/miR-145-5p/FAO axis in cancer cells	[74]
In vitro	Human BM	–	Breast cancer	Strengthening cancer cells expansion	Inducing the regulation of LINC01133 in neighboring tumor cells	[75]
In vitro and in vivo	Rat BM	Rats of the F1 cross between the inbred strains BN and WF with colon carcinoma	Colon carcinoma	Inducing anticancer immunity	Secreting several mediators to recruit massive inflammatory cells to tumor site	[76]
In vitro and in vivo	Human BM	nude mice with KSIMM tumor	Kaposi’s sarcoma	Exerting potent antitumorigenic effects	Inhibiting target cells Akt activity	[77]
In vitro and in vivo	Human dermis tissues of a dead fetus	SCID mice with MCF-7 breast carcinoma	Breast cancer	Inhibiting breast cancer progression	Secreting Dkk-1 to inhibit breast cancer progression via depression of Wnt signaling	[78]
In vitro and in vivo	Murine BM	BALB/c mice with H22 tumor	HCC	Inhibiting cancer	Inducing tumor cell apoptosis and G0/G1 phase arrest	[79]
In vitro and in vivo	The BM of Sprague–Dawley rats and C57BL/6 J mice	C57BL/6 J mice with B16F10 tumor	Melanoma	Antiangiogenesis	Migrating toward the capillaries, intercalating between ECs, establishing Cx43-based intercellular GJC with ECs, and increasing production of ROS	[80]
In vitro and in vivo	Human BM	BALB/c nude mice with Hep3B-CSCs tumor	Hepatocellular carcinoma	Blocking malignant behaviors of hepatocellular CSCs	Working through a lncRNA C5orf66‑AS1/microRNA‑127‑3p/DUSP1/ERK axis	[81]

*MIP-2* macrophage inflammatory protein-2; *VEGF* vascular endothelial growth factor; *ECs* endothelial cells; *ROS* reactive oxygen species; *GJC* gap junctional communication; *AT* adipose tissue; *BM* bone marrow; *WF* Wistar/Furth; *DUSP1* dual-specificity phosphatase 1

### MSCs inhibit cancer progression

Notwithstanding the effects of MSCs on cancer progression, studies have demonstrated their inhibitory effect on tumor growth. As a proinflammatory factor, the combination of MSCs and tumor cells increased the infiltration of monocytes, granulocytes as well as T lymphocytes. The increased infiltration of inflammatory cells creates opportunities for cross-talk between these immune cells and the surrounding tissues. These immune cells, as well as the surrounding inflamed tissues, can generate several chemokines that attract activated lymphocytes with the corresponding receptors, thereby further inducing anticancer immunity [76]. Furthermore, Aarif and colleagues showed that MSCs suppressed tumor growth in vivo by inhibiting target cell AKT activity in Kaposi’s sarcoma (KS). However, they discovered that when KS tumor cells were engineered to express active AKT continuously, KS tumors were no longer sensitive to MSCs administration. Their findings suggest that MSCs exert potent antitumorigenic effects by inhibiting AKT signaling [77]. Similarly, Qiao et al. demonstrated that MSCs suppress breast cancer cell proliferation via the Wnt pathway, which is crucial in tumourigenesis [78] (Fig. 1). In addition, in a study by Lu and colleagues, they showed that MSCs administration enhanced the mRNA expression of p21 (cell cycle negative regulator) and caspase 3 (apoptosis associated protease) in tumor cells. Their findings demonstrated that MSCs can inhibit cancer progression in vitro and in vivo by increasing apoptosis and G0/G1 phase arrest in cancer cells [79]. Furthermore, MSCs have been shown to suppress cancer by inhibiting tumor angiogenesis through endothelial cells apoptosis and capillary degeneration [80]. Recently, Gu and colleagues reported that MSCs‑derived Exo were able to block hepatocellular CSCs malignancy via a lncRNA C5orf66‑AS1/ microRNA‑127‑3p/dual-specificity phosphatase 1 (DUSP1)/ERK axis [81]. Considering that Exo are involved in both oncogenic and tumor-suppressing roles of MSCs, they treated hepatocellular CSCs with MSC-Exo, and found that the proliferation, migration, invasion, angiogenesis-stimulating and self-renewal abilities of CSCs were significantly decreased through lncRNA C5orf66‑AS1/microRNA‑127‑3p/DUSP1 axis and inhibiting the phosphorylation of ERK in vitro. And in vivo, similar results were observed, which showed that Exo attenuated the growth of xenograft formed by CSCs in nude mice. Their study may offer novel insights into the relevance of MSCs and their derived molecules to cancer progression, and in particular, to the stem cell property of CSCs (Table 1).

Alternatively, engineered MSCs are gaining popularity because of their propensity to migrate to tumor locations. For example, TNF-related apoptosis-inducing ligand (TRAIL), nanoparticles, bone morphogenetic protein 4 (BMP4) and other molecules that can limit cancer cell development were used for modifying MSCs, which reduced cancer cell growth and metastasis while also causing apoptosis [82–84]. More molecules were administered to engineered MSCs, as shown in Table 3. These findings indicated that modified MSCs can reduce the proliferation and migration of cancer cells in vitro and in vivo, implying that MSCs could become a potential therapeutic for cancer.

## MSCs in anticancer therapeutic resistance

Resistance to therapeutic therapy leads to the limited efficacy of cancer treatments. Although emerging evidence indicates that tumor cell-intrinsic gene alterations contribute to therapeutic resistance, an increasing number of reports have suggested that the TME is also crucial in the evolution of therapeutic resistance [85]. Intrinsic resistance develops over time, while external resistance can be mediated by signaling or soluble molecules from the TME. The latter resistance may be reversible because therapeutic sensitivity was restored when these TME mediators were removed [85]. Thus, it is crucial to understand the mechanism of external therapeutic resistance. Notably, MSCs, which are an important part of the TME, are known to play a key role in the resistance of cancer treatment [86].

### MSCs promote therapeutic resistance

More studies showed that tumor growth was promoted in the presence of MSCs in the TME, thus indicating that MSCs promote resistance to therapy, which is included among hematological malignancies. For example, in chronic myeloid leukemia (CML), MSCs protected CML cells from imatinib-induced cell death via the CXCL12/CXCR4 axis, which decreased caspase 3 activity [87]. Similarly, in chronic lymphoid leukemia (CLL), forodesine-induced CLL cell apoptosis was antagonized by MSCs, which inhibited forodesine-induced RNA and protein synthesis activity and increased the expression of the antiapoptotic protein Mcl-1 at both the transcript and protein levels [88]. Moreover, in multiple myeloma (MM), it was found that MSCs enhanced MM cell resistance to bortezomib through CXCL13 production [89]. In solid tumors, MSCs have also been shown to promote resistance to therapeutic therapy. For instance, in head and neck carcinoma, MSCs were reported to increase chemoresistance to paclitaxel by secreting several mediators, such as IL-7, IL-8, IGF and EGF [90]. In ovarian cancer, tumor-related MSCs were able to induce resistance to hyperthermic intraperitoneal chemotherapy by activating the CXCL12-CXCR4 axis, and when the CXCL12/CXCR4 interaction was blocked, the cytotoxicity of hyperthermia was restored [91]. In addition, the expression of IL-6 produced by MSCs is another key mechanism of resistance. IL-6 raised the levels of Bcl-2 and Bcl-X_L_, which inhibited cancer cell apoptosis after cytotoxic therapy [92]. Moreover, MSC-secreted IL-6 has been shown to enhance tumor resistance by increasing the formation of CSCs [57]. In colorectal cancer, tumor cells release IL-1, which induces MSCs to secrete PGE_2_ and raises IL-6 level, which leads to the development of CSCs [93]. In addition to paracrine mechanism, the process of systematical secretion by MSCs is also implicated in the promotion of therapeutic resistance. Recently, Roodhart et al. showed that endogenous MSCs exposed to cisplatin became activated to protect tumor cells against chemotherapeutics through systematically secreting two different platinum-induced polyunsaturated fatty acids (PIFAs), 12-oxo-5,8,10-heptadecatrienoic acid (KHT) and hexadeca-4,7,10,13-tetraenoic acid [16:4(n-3)]. Both PIFAs induced resistance to a range of chemotherapeutic agents in minute quantities. Moreover, the author discovered that blocking key enzymes implicated in PIFA synthesis (such as cyclooxygenase-1 and thromboxane synthase) inhibited MSC-induced resistance [94]. Their findings revealed that MSCs may generate chemoresistance even when they are not from tumors, and that MSCs can function as strong mediators of therapeutic resistance (Fig. 1).

In addition to secreting mediators, MSCs have the ability to transform into CSCs or provide CSC niche support. CSCs are a type of cancer cells that reside in tumor tissues at a low level, and they are resistant to many cytotoxic agents, partly because of their low proliferative rate, high level of membranal transporters that could control cytotoxic drug influx, and high DNA repair ability [95, 96]. Here, we will discuss how MSCs contribute to therapeutic resistance through this mechanism. Several tumor suppressor genes in MSCs, such as ras-associated family protein isoforms 1A (RassF1A) and hypermethylated in cancer 1 (HIC1), were methylated, which causes MSCs to convert into CSC cells and demonstrates MSCs’ tumorigenic potential. In vitro, MSCs which were targeted methylations of HIC1/RassF1A lost anchorage dependence, and increased drug resistance, colony formation capability and pluripotency. In animal model, minimal quantities of targeted MSCs were injected in immunodeficient mice, resulting in tumor formation. These findings suggested that MSCs might convert into CSCs, increasing chemoresistance and allowing tumor recurrence after treatment cessation [97]. Moreover, MSCs were indicated to regulate CSCs to facilitate tumor growth and chemoresistance. In pancreatic ductal adenocarcinoma (PDAC), MSCs exposed to gemcitabine significantly enhanced CXCL10 expression, which further promoted CSC proliferation by overexpressing the CXCL10 receptor (CXCR3) on CSCs. As a result, tumor growth and chemoresistance were both boosted [98] (Fig. 1). Furthermore, MSCs pretreated with cisplatin altered the phosphorylation state of several tyrosine kinases, such as WNK1, c-Jun, STAT3 and p53, and produced factors that turn on the changes in stemness and resistance of tumor cells, thus promoting therapeutic resistance of tumor cells [99] (Table 2). However, it is not clear how MSCs and these mediators contribute to therapeutic resistance.

**Table 2:** Roles of MSCs in anticancer therapeutic resistance

In vitro or in vivo	Source of MSCs	In vivo model	Cancer types	Functions	Mechanisms	References
In vitro and in vivo	Human BM	NOD/SCID mice with BV173 tumor	CML	Nonselectively protecting CML cells	Protecting CML cells from imatinib-induced cell death via the CXCL12/CXCR4 axis	[87]
In vitro	Murine and human BM	–	CLL	Antagonizing forodesine-induced CLL cell apoptosis	Inhibiting forodesine-induced RNA- and protein-synthesis activity and increasing the expression of Mcl-1	[88]
In vitro	Human BM	–	MM	Enhancing MM cells’ resistance to bortezomib	Secreting CXCL13	[89]
In vitro	Human BM	–	HNC	Increasing chemoresistance to paclitaxel	Secreting several mediators, such as IL-6, IL-7, IL-8, IGF and EGF	[90]
In vitro	TA-MSCs isolated from ascites of ovarian cancer patients and human BM	–	Ovarian cancer	Inducing the resistance of HIPEC	Activating the CXCL12-CXCR4 axis	[91]
In vivo	Human and murine BM	BALB/c mice with C26 tumor, C57BL/6 mice with LLC tumor and nude mice with MDA-231-MB tumor	Colon carcinoma, lung cancer, breast cancer	Protecting tumor cells against chemotherapeutics	Systematically secreting two different PIFAs	[94]
In vitro and in vivo	Human BM	Nude mice with control or methylated MSCs	Soft tissue sarcomas-like	Promoting resistance to cisplatin and contributing to tumor relapse	Transforming into CSCs	[97]
In vitro and in vivo	Human BM	NOD/SCID mice with SUM159 tumor	Breast cancer	Regulating CSCs to accelerate tumor growth	Secreting several cytokines, such as CXCL7 and IL-6	[57]
In vitro and in vivo	Human BM	Nude mice with LoVo tumor	Colorectal cancer	Inducing the formation of CSCs	Tumor cells released IL-1 which induced MSCs to secrete PGE2 and increase the level of IL-6, IL-8 and CXCL1	[93]
In vitro and in vivo	Human BM	SCID mice with PANC1 or A549 tumor	PDAC; lung cancer	Increasing tumor growth and enhancing chemoresistance	Increasing CXCL10 expression	[98]
In vitro and in vivo	Human AD	Nude mice (BALB/c-nu/nu) with MDA-MB-231 tumor	Breast cancer	Inducing chemoresistance of tumor cells	Altering phosphorylation state of several tyrosine kinases, such as WNK1, c-Jun, STAT3 and p53	[99]
In vitro and in vivo	Human UC	Nu/Nu nude mice with MDA-MB-231 tumor	Breast cancer	Suppressing breast cancer pathogenesis and sensitizing cancer cells to radiotherapy	Blocking STAT3 pathway and decreasing CSCs’ stemness	[102]

*UC* umbilical cord; *TA* tumor-associated

### MSCs inhibit therapeutic resistance

In addition to promoting therapeutic resistance, MSCs were reported to increase sensitivity to therapies. The presence of CSCs in tumor tissues is the most common cause of therapeutic resistance and disease relapse [100], and STAT3 contributes to the maintenance of CSCs programs in cancer cells [101], so He and colleagues examined the expression of several stemness genes related to CSCs (Sox2, Oct4, Nanog, and c-Myc) as well as STAT3 signaling-related genes (p53, cyclin D1, Bcl-X_L_) in MDA-MB-231 cells. Their results showed that the levels of these genes were significantly reduced in the MSCs-CM and the combination groups (radiation combined with MSC therapy). Thus, they concluded that MSCs could suppress breast cancer pathogenesis and sensitize cancer cells to radiotherapy through down-regulating the STAT3 pathway, providing a novel therapeutic for breast cancer in terms of overcoming radioresistance [102] (Table 2) (Fig. 1). In addition, engineered-MSCs were able to inhibit therapeutic resistance (Table 3). However, because the number of relevant studies is limited, additional researches are needed to fully understand the role of MSCs in therapeutic resistance.

**Table 3:** Antitumor molecules delivered by MSCs

Molecules	Functions	Cancer types	References
IL-2	Delaying tumor growth and developing CD8-mediated tumor-specific anticancer immunity	Melanoma	[114]
IL-12	Boosting antitumor T cell responses and inhibiting tumor growth	Melanoma; cervical cancer	[113]
IL-18	Activating T cells	GBM	[143]
NK4	Inhibiting angiogenesis and promoting apoptosis	Lung cancer	[144]
IFN-β	Inhibiting the growth of cancer cells	Melanoma; metastatic prostate cancer	[115, 145]
IFN-γ	Immunostimulation and stimulating apoptosis	Leukemia	[146]
CX3CL1	Activating CD8^+^ and NK cells	Lung cancer	[147]
PE-cytotoxins	–	GBM	[148]
sFlt-1	Inhibiting angiogenesis and metastasis	Tumor lung metastasis	[149]
iNOS	Inhibiting growth of tumor cells	Fibrosarcoma	[150]
HSV-TK	Transferring ganciclovir into active cytotoxic drugs	GBM	[112]
Nanoparticle	Drug-loaded polymeric nanoparticles	Lung cancer	[83]
TRAIL	Inducing apoptosis	Lung cancer	[82]
Cytosine deaminase	Converting inactive systemically administered prodrugs into active cytotoxic agents	Colon cancer; prostate cancer	[110, 111]
rCE	Converting the CPT-11 to SN-38	GBM	[151]
miR-124a	Decreasing survival of cancer cells	GBM	[152]
miR-143	Inhibiting cancer migration	Osteosarcoma	[153]
miR-146b	Inhibiting cancer cells growth	GBM	[154]
Cytotoxic chemotherapy agents	Inhibiting cell viability and reducing cancer cell growth	Oral squamous cancer	[128]
miR-193a	Reducing cisplatin resistance of NSCLC cells by downregulating LRRC1	NSCLC	[131]
anti-miR-9	Reversing the chemoresistance of GBM cells to TMZ by regulating the expression of multidrug transporter	GBM	[132]
siGRP78	Reversing the drug resistance	HCC	[133]
miR-122	Enhancing cancer cells chemosensitivity to sorafenib by downregulating the expression of CCNG1, ADAM10 and IGF1R	HCC	[134]
MiR-6785-5p	Suppressing angiogenesis and metastasis in GC	GC	[155]

*NK4* HGF antagonist/angiogenesis inhibitor; *PE-cytotoxins* pseudomonas exotoxin-cytotoxins; *sFlt-1* soluble fms-like tyrosine kinase 1; *iNOS* inducible nitric oxide synthase; *rCE* rabbit carboxylesterase; *NK cells* natural killer cells; *GC* gastric cancer

## MSCs: a double-edged sword

MSCs were reported to have pro- and anti-cancer effects. The opposing effects may depend on several factors. For example, the roles of MSCs in cancer progression differ depending on the tumor model employed. In an in vivo model of KS, bone marrow derived MSCs (BMSCs) administered intravenously (i.v.) home to carcinogenesis sites and potently suppress tumor growth. And in vitro, co-culture of MSCs with KS cells inhibit the proliferation of cancer cells. This effect of inhibiting tumor growth requires the BMSCs to achieve direct cell–cell contact via blocking the activation of Akt signaling [77]. In contrast, in an in vivo model of osteosarcoma, BMSCs injected i.v. targeted the osteosarcoma site and encouraged its growth and pulmonary metastasis, and in vitro, osteosarcoma cells’ proliferation was enhanced in the presence of BMSC-CM, suggesting a contact-independent mechanism [103]. Moreover, the differentiation degree and source of MSCs also affect MSCs’ functions. For example, adipocyte-differentiated MSCs significantly reduced all-trans retinoic acid- and doxorubicin- induced apoptosis of acute promyelocytic leukemia cells in vitro. MSCs’ protective properties were attributed to the synthesis of leptin by adipocyte-differentiated MSCs. This antiapoptotic action of leptin required the stimulation of STAT3 and MAPK signaling [104]. Similarly, osteoblasts differentiated from MSCs increased acute myelogenous leukemia (AML) cell engraftment of in BM and protected AML cells from chemotherapy-induced death [105]. On the other hand, MSCs derived from human umbilical cord blood and adipose tissue induced apoptosis of breast cancer cells by enhancing PARP and caspase-3 cleavage [106]. In addition, it was revealed that MSC functions differ in vitro and in vivo, and this difference has been demonstrated in various cancer cell lines. MSCs showed an antiproliferative role by causing G1 arrest in vitro; however, mice injected with cancer cells and MSCs exhibited faster tumor growth in vivo [107]. These results exposed a significant roadblock in the process of putting anticancer MSCs into clinical therapy. Despite the fact that numerous studies have demonstrated that MSCs have a protumorigenic effect, they were genetically engineered as a vehicle to target cancer cells. This method has been proven to be effective in multiple cancer models [82–84](Table 3). In summary, MSCs are a group of pluripotent cells that may migrate to tumor sites and have a positive or negative impact in cancer progression via different mechanisms.

## MSCs as a potential therapeutic avenue for cancer

MSCs’ immunosuppressive and regenerative abilities suggest that they might be used to treat a variety of illnesses. Meanwhile, MSCs have been chosen as drug delivery vehicles in a variety of cancers because of their propensity to homing to tumor locations. For example, MSCs were used to deliver oncolytic viral loads into tumors, thereby effectively killing cancer cells [108, 109]. Moreover, MSCs were genetically engineered to express specific enzymes, such as herpes simplex virus-thymidine kinase (HSV-TK) and cytosine deaminase, which have the ability to convert inactive systemically administered prodrugs into active cytotoxic agents, thus further enhancing chemotherapy sensitivity and reducing potential toxicity [110–112]. These data suggested that MSCs were efficient anticancer delivery agents, improving tumor killing specificity and decreasing systemic toxicity.

Another method for designing MSCs is that MSCs continuously generate unique immunomodulatory cytokines that can induce cancer cell death. For example, MSCs with IL-12 overexpression enhanced antitumor T cell responses and inhibited tumor growth [113]. In melanoma, IL-2-expressing MSCs were indicated to delay tumor growth, developing CD8-mediated tumor-specific anticancer immunity [114]. In melanoma and metastatic prostate cancer, IFNβ-expressing MSCs inhibited the growth of cancer cells [115]. Moreover, MSCs with genetic alterations can directly target cancer cells. For instance, TRAIL-expressing MSCs have been shown to effectively kill cancer cells in multiple cancer models, such as lung, glioblastoma (GBM), pancreatic and colorectal cancers [116–120]. In addition, TRAIL-expressing MSCs can directly target CSCs in lung cancer, reducing tumor aggressiveness and chemoresistance as well as relapse [121]. Furthermore, MSCs were also utilized to treat residual disease that has undergone chemotherapy, radiation, or surgery. In GBM, MSCs engineered with TRAIL and/or oncolytic viruses were able to effectively kill residual tumor cells [122, 123]. More data have been included in Table 3. Taken together, genetically modified MSCs appear to be a potential cancer treatment option.

However, MSC-based cell treatment may have several potential drawbacks, including noneffective local concentrations of drugs within tumors and nonspecific dissemination throughout the tissue and organism [124]. In addition, their physiological function of differentiating into mesenchymal lineages may enhance immunogenicity, lower therapeutic potential, and promote tumorigenesis [125]. To overcome these obstacles, extracellular vehicles (EVs) generated from MSCs have been disclosed as a drug delivery strategy for killing cancer cells. Similar to parental MSCs, such EVs still have tumor homing ability [126] and immune-suppressive properties [127]. Emerging evidence indicated that EVs were designed to overexpress cytotoxic chemotherapy agents, selectively killing cancer cells. For example, in oral squamous cancer, paclitaxel-, doxorubicin- or gemcitabine-expressing EVs inhibited cell viability and reduced cancer cell growth [128] (Table 3). Moreover, in the same cancer type, MSCs are also implicated in the composition of M/LPV/O_2_, which increases targeting efficacy and overcomes tumor hypoxia-associated resistance in sonodynamic therapy, thus enhancing therapeutic outcomes [129]. In addition, Exo, a kind of EVs, has been linked to cancer progression. Exo are enclosed vesicles with small membranes that communicate with other cells. They can load several molecules, including lipids, proteins, nucleic acids, mRNA and miRNA [130]. For example, in non-small cell lung cancer (NSCLC), Wu et al. found that exosomal miR-193a in MSCs reduces the cisplatin resistance of NSCLC cells by downregulating leucine-rich repeat-containing protein 1 (LRRC1), thus providing new insights into a novel therapeutic for NSCLC [131]. In contrast to Wu’s work, Jessian and colleagues performed anti-miR delivery by MSC-derived Exos to explore the chemosensitivity of GBM cells. Their findings demonstrated that the delivery of MSC-Exo-anti-miR-9 to cancer cells reversed the chemoresistance of GBM cells to temozolomide by regulating the expression of multidrug transporters. Their report revealed a particular target for GBM via anti-miR delivery in the RNA therapeutics field [132]. Similarly, in hepatocellular carcinoma (HCC), Li et al. manipulated MSCs to express exosomal siGRP78, which can target GRP78 overexpressed in resistant HCC cells. Their findings suggested that siGRP78-modified MSC-Exos might sensitize sorafenib-resistant HCC cells to sorafenib to reverse drug resistance [133]. Additionally, in HCC, Lou et al. indicated that MSCs Exos promoted miR-122-enhanced HCC cell chemosensitivity to sorafenib in HCC cells by downregulating the expression of miR-122 target genes, such as cyclin G1 (CCNG1), disintegrin and metalloprotease 10 (ADAM10), and insulin-like growth factor receptor 1 (IGF1R), which are involved in the drug sensitivity or resistance of cancer cells. Their study supported that MSC exosome-miR-122 is a novel strategy to increase HCC chemosensitivity [134] (Table 3). In PDAC, paclitaxel (PTX) and gemcitabine monophosphate (GEMP) were loaded in/on BMSC Exos in Zhou’s study. The “Exo” platform they constructed was capable of overcoming chemoresistance because it had a tumor-homing property to overstep the barriers of pathological extracellular matrix to further enhance the accumulation of PTX and GEMP in the tumor site [135]. Based on combined studies, the MSC delivery system may serve as a promising strategy for inhibiting therapeutic resistance in cancer treatment.

Nevertheless, long-term cultures of MSCs for treatment were shown to commonly undergo spontaneous malignant transformation, with transformed mesenchymal cells leading to tumors in vivo [136]. Then after 5 years, the initial report was retracted because the authors were unable to replicate some of the reported spontaneous transformation events and suspected that the phenomenon is due to a cross-contamination artifact [137]. These findings highlight the need of following strict cell culture methods when it comes to medicinal reasons. Moreover, genetic abnormalities were also observed in vitro [138]. Notably, there are no clear evidence that chromosomal changes lead to malignant transformation in vitro or in vivo [139]. In addition, an examination of tissues following MSC treatment in humans revealed no evidence of malignant tumors originating from MSCs [140]. Even yet, we can’t rule out the possibility of malignancies forming after MSC therapy since chromosomal abnormalities in MSCs may occur at the time of injection or afterward. More patient follow-up research on MSC therapy should be required. Out of an abundance of caution, standardized purification and expansion protocols must be created, given the possibility of chromosomal aberrations under culture conditions [141]. As a result, culture conditions with low proliferation rates and limited expansion rates have been proposed to reduce the likelihood of acquired chromosomal abnormalities [141].

Generally, the combined treatments of MSCs and other existing treatment modalities have proven to be a promising therapeutic in several cancer types, and simultaneously, uniform purification and expansion protocols in vitro must be followed.

## Conclusions

The roles of MSCs in cancer progression and anticancer therapeutic resistance are of versatility and plasticity. MSCs have been implicated in promoting cancer, including promoting the survival, metastasis, angiogenesis and evasion of the immune system, as well as inhibiting apoptosis. MSCs facilitate cancer progression and therapeutic resistance by close interactions with cancer cells or systemic/paracrine mechanisms involving secreted factors. MSC-targeting treatment might represent an anticancer therapy and improve therapeutic sensitivity. MSCs show diverse mechanisms of enhancing cancer progression in vitro; however, whether MSCs play the same roles in vivo has been undetermined. As a result, inhibiting MSCs in a clinical setting becomes a challenge. The ability of MSCs homing to tumor sites aids in the precise targeting of malignancies. However, it is worth noting that these genetically engineered MSCs may have inherent mechanisms that facilitate cancer cell proliferation, metastasis and therapeutic resistance, implying that engineered MSCs used to target tumors might have unintended consequences if handled inappropriately. Although numerous studies have shown that MSCs have cancer-promoting properties, several publications have shown that MSCs can also inhibit cancer progression. Different stimuli on MSCs depend on the MSC status and have positive or negative effects on cancer etiology. However, the precise mechanisms of these phenomena are still not clear. In summary, MSCs have key effects on tumor growth and treatment response; thus, MSCs alone or in combination with other treatments may become a promising treatment for cancer. To address the clinical challenges outlined above, additional researches should be required.

## Data Availability

Not applicable.

## Abbreviation

MSCs: Mesenchymal stem cells
BM: Bone marrow
TME: Tumor microenvironment
TLR: Toll-like receptors
PGE_2_: Prostaglandin E_2_
IDO: Indoleamine 2,3-dioxygenase
GARP: Glycoprotein a repetitions predominant
Treg: Regulatory T cells
CM: Conditioned medium
FAO: Fatty acid oxidation
KS: Kaposi’s sarcoma
DUSP1: Dual-specificity phosphatase 1
TRAIL: TNF-related apoptosis-inducing ligand
BMP4: Bone morphogenetic protein 4
CML: Chronic myeloid leukemia
CLL: Chronic lymphoid leukemia
MM: Multiple myeloma
PIFAs: Platinum-induced polyunsaturated fatty acids
CSCs: Cancer stem cells
RassF1A: Ras-associated family protein isoforms 1A
HIC1: Hypermethylated in cancer 1
PDAC: Pancreatic ductal adenocarcinoma
BMSCs: Bone marrow derived MSCs
AML: Acute myelogenous leukemia
GBM: Glioblastoma multiforme
EVs: Extracellular vehicles
SDT: Sonodynamic therapy
NSCLC: Non-small cell lung cancer
LRRC1: Leucine-rich repeat-containing protein 1
HCC: Hepatocellular carcinoma
PTX: Paclitaxel
GEMP: Gemcitabine monophosphate
CAF: Cancer-associated fibroblasts
EMT: Epithelial-mesenchymal transition
